# A Xanthohumol-Rich Hop Extract Diminishes Endotoxin-Induced Activation of TLR4 Signaling in Human Peripheral Blood Mononuclear Cells: A Study in Healthy Women

**DOI:** 10.3390/ijms232012702

**Published:** 2022-10-21

**Authors:** Finn Jung, Raphaela Staltner, Anja Baumann, Katharina Burger, Emina Halilbasic, Claus Hellerbrand, Ina Bergheim

**Affiliations:** 1 Department of Nutritional Sciences, Molecular Nutritional Science, University of Vienna, Josef-Holaubek Platz 2, 1090 Vienna, Austria; 2 Department of Medicine III, Medical University of Vienna, Währinger Gürtel 18-20, 1090 Vienna, Austria; 3 Institute of Biochemistry, Friedrich-Alexander University Erlangen, 91054 Erlangen, Germany

**Keywords:** CD14, LPS, hop, TLR4, inflammation

## Abstract

Infections with Gram-negative bacteria are still among the leading causes of infection-related deaths. Several studies suggest that the chalcone xanthohumol (XN) found in hop (Humulus lupulus) possesses anti-inflammatory effects. In a single-blinded, placebo controlled randomized cross-over design study we assessed if the oral intake of a single low dose of 0.125 mg of a XN derived through a XN-rich hop extract (75% XN) affects lipopolysaccharide (LPS)-induced immune responses in peripheral blood mononuclear cells (PBMCs) ex vivo in normal weight healthy women (*n* = 9) (clinicaltrials.gov: NCT04847193) and determined associated molecular mechanisms. LPS-stimulation of PBMCs isolated from participants 1 h after the intake of the placebo for 2 h resulted in a significant induction of pro-inflammatory cytokine release which was significantly attenuated when participants had consumed XN. The XN-dependent attenuation of proinflammatory cytokine release was less pronounced 6 h after the LPS stimulation while the release of sCD14 was significantly reduced at this timepoint. The LPS-dependent activation of hTLR4 transfected HEK293 cells was significantly and dose-dependently suppressed by the XN-rich hop extract which was attenuated when cells were co-challenged with sCD14. Taken together, our results suggest even a one-time intake of low doses of XN consumed in a XN-rich hop extract can suppress LPS-dependent stimulation of PBMCs and that this is related to the interaction of the hop compound with the CD14/TLR4 signaling cascade.

## 1. Introduction

Despite a large selection of antibiotics, infections with Gram-negative bacteria are still among the leading causes of morbidity and mortality in the world, also as antibacterial multidrug resistance (AMR) is constantly increasing. Indeed, it has been estimated that in 2019 4.95 million deaths worldwide were associated with AMR. From those, 2.2 million deaths were related with AMR against the four most frequent Gram-negative bacteria *Eschericia coli, Klebsiella pneunomoniae, Acinetobacter baumannii* and *Pseudomonas aeruginosa* [1]. Furthermore, results of studies in patients with metabolic diseases, e.g., type 2 diabetes and non-alcoholic fatty liver disease (NAFLD) but also in patients with alcohol-related liver disease suggest that Gram-negative bacteria and even more so components of their outer cell wall, e.g., lipopolysaccharides (LPS), may also be an important trigger in the development of these diseases [2]. For instance, it has been shown that the loss or blockage of toll-like receptor 4 (TLR4), attenuates the development of NAFLD and alcohol-related liver diseases and improves insulin signaling in settings of type 2 diabetes [3,4,5]. TLR4 has been shown to bind bacterial LPS through a complex interplay of cluster of differentiation 14 (CD14) and myeloid differentiation factor 2 (MD-2) [6]. Furthermore, in macrophages and monocytes the LPS-dependent activation of TLR4 signaling has been shown to result in a marked induction of cytokines such as interleukin-1β (IL-1β), interleukin-6 (IL-6) and tumor necrosis factor α (TNF-α) [7,8].

Results of in vivo and in vitro studies suggest that secondary plant compounds found in hop-like α-acids and β-acids as well as xanthohumol (XN) may possess anti-inflammatory effects in various disease settings [9]. For example, despite having been suggested to be poorly absorbed [10], XN has been shown to possess anti-inflammatory effects in the development of NAFLD and insulin resistance but upon stimulation with lipoteichoic acid derived from Gram positive bacteria [11,12]. Furthermore, XN has been suggested to interfere with the TLR4 signaling cascades [13,14,15]. Specifically, in in vitro cell and molecular docking studies it has been suggested that XN may suppress endotoxin-induced TLR4 activation through interfering with endotoxin binding to MD-2 [13,16]. However, molecular mechanisms underlying the beneficial effects of XN on inflammatory processes induced by LPS are still not fully understood. In addition, the doses of XN used in most studies were within the pharmacological range, i.e., 12 mg/d per person or higher [17,18]. If XN, when ingested at low doses (as they could be found in approximately 250 mL beer (XN concentrations vary between 0.002 and 0.69 mg/L, depending on the type of beer [19,20]), also exerts beneficial effects in humans, e.g., attenuates the inflammatory responses triggered by Gram-negative bacteria or LPS, has not yet been assessed.

In the present study, we therefore aimed to determine if, in healthy, normal weight women, the intake of a low dose of XN (0.125 mg XN derived through a XN-rich hop extract (75% XN), dose based on concentrations found in 250 mL beer [19,20] contained in a beverage consumed along with a light breakfast affects LPS-dependent immune response of peripheral blood mononuclear cells (PBMCs) isolated after the intake of the hop compound. Furthermore, molecular mechanisms underlying the effects of XN on TLR4 signaling cascade were assessed.

## 2. Results

### 2.1. Co-Culture Cell Assay

**Figure 1:**
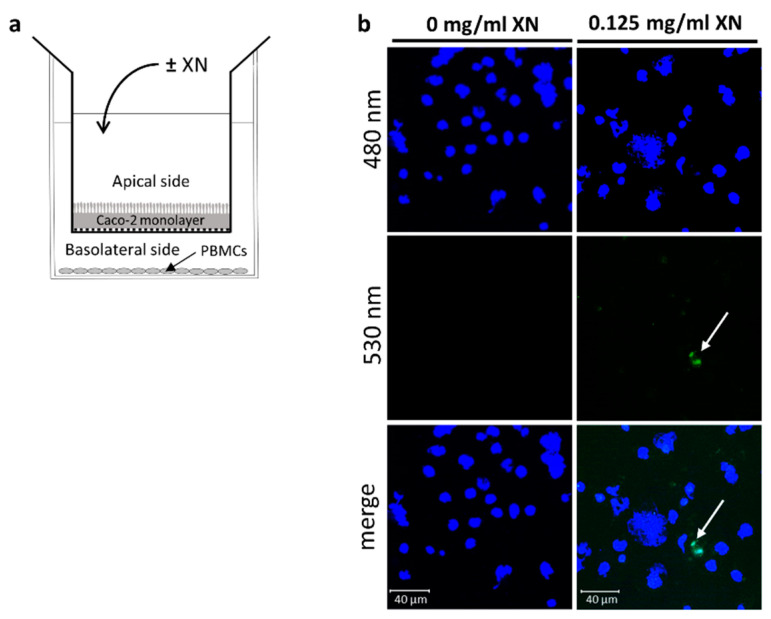
Fluorescence imaging and XN determination of PBMCs isolated from buffy coat of healthy donors co-cultured with Caco-2 cells incubated with XN. (**a**) Graphical illustration of the experimental co-culture setup using Caco-2 cells and PBMCs. (**b**) Representative pictures of fluorescence of XN in PBMCs cells after incubation of Caco-2 cells ± XN (0.125 mg/mL derived through a XN-rich hop extract) for 1 h (magnification 400×) in a co-culture model. White arrows indicate autofluorescence of XN. PBMC, peripheral blood mononuclear cell, XN, xanthohumol. Data are expressed as means ± SEM.

To determine if XN passes the intestinal barrier and binds or is taken up by blood immune cells, a co-culture model, consisting of differentiated Caco-2 cells and isolated human PBMCs obtained from buffy coats, was employed (for experimental design see Figure 1a). PBMCs were spotted on glass slides and counterstained with DAPI (480 nm). One hour after the exposure to 0.125 (Figure 1b), 0.375 and 0.750 mg/mL XN (data not shown), respectively, autofluorescence of XN at 530 nm was detected in PBMCs seeded in the basolateral compartment of the transwell system (indicated with white arrow) whereas no fluorescence was detected at 530 nm in untreated cells. Merged pictures indicate that XN is either bound or taken up by PBMCs. Based on these results, it was concluded that that XN is rapidly taken up/passes through enterocytes and an exposure time of 1 h was selected for the human intervention.

### 2.2. Effect of the Oral Intake of XN on LPS-Induced Inflammation

**Figure 2:**
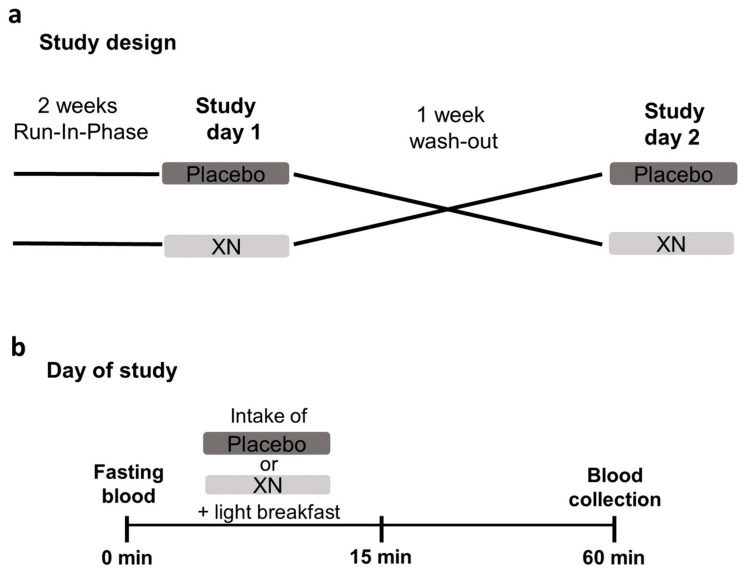
Graphical visualization of the study design. (**a**) Study design and (**b**) the procedure performed on each day of the study. XN, xanthohumol derived though a xanthohumol-rich hop extract.

Of the 12 normal weight, healthy women enrolled in the study, 9 were analyzed. The study design is summarized in Figure 2a,b. Three women had to be excluded from the analysis as, for some time points, the numbers of cells obtained were not sufficient for the stimulation experiments. Characteristics of the analyzed participants and routine laboratory parameters are shown in Table 1.

**Figure 3:**
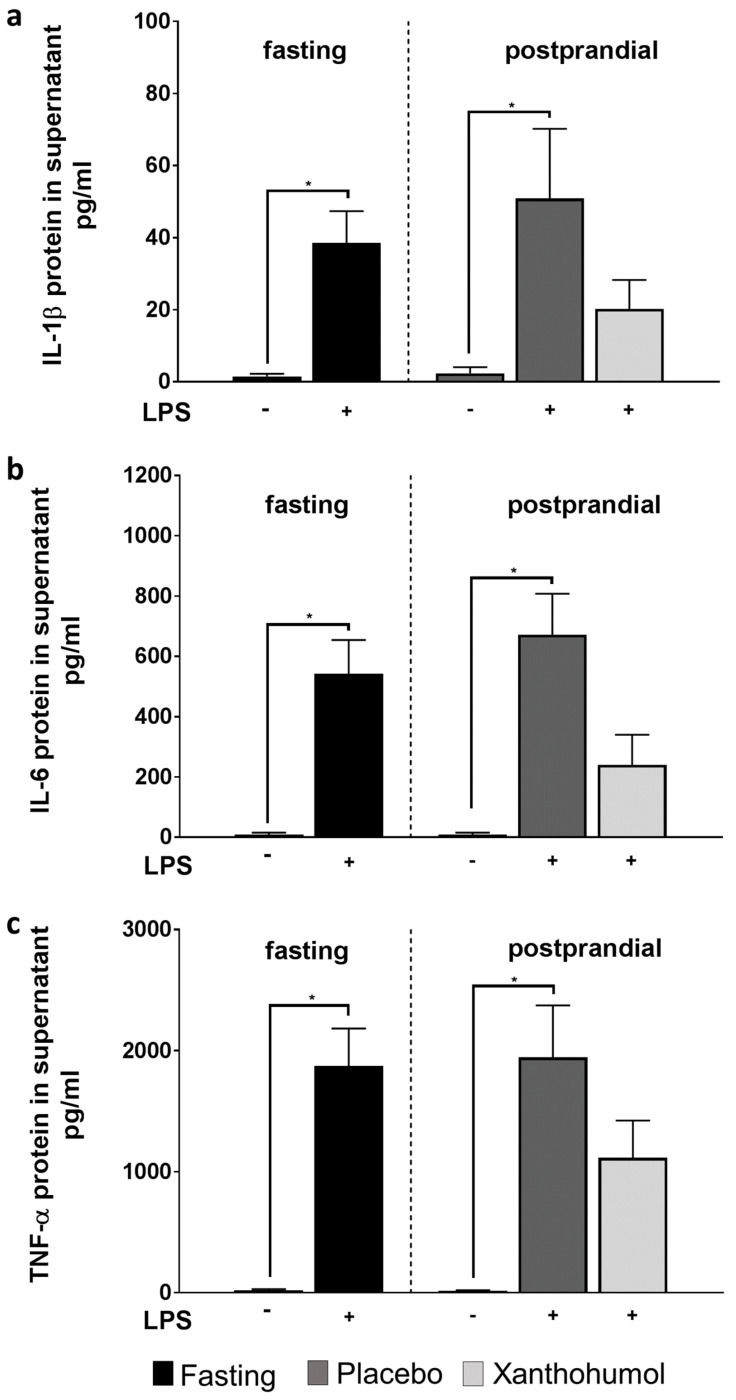
Cytokine concentrations in supernatant of LPS−stimulated PBMCs obtained from healthy study participants. Protein concentrations of IL−−1β (**a**), IL−−6 (**b**) and TNF−−α (**c**) in cell culture supernatant of PBMCs stimulated with 0 or 100 ng/mL LPS for 2 h isolated from healthy study participants receiving either a placebo or the study drink containing XN derived through a XN−rich hop extract. IL, interleukin; LPS, lipopolysaccharide; PBMC, peripheral blood mononuclear cell; XN, xanthohumol. Data are expressed as means ± SEM. * = *p* < 0.005.

As expected, after a 2 h challenge with LPS, protein levels of IL-1β, IL-6, and TNF-α in cell supernatant of PBMCs isolated before the intake of the beverages were significantly higher than in cells only treated with plain cell culture media. In line with these findings, in cell supernatant of LPS-treated PBMCs isolated after the intake of the placebo, protein levels of the three pro-inflammatory cytokines were also significantly higher than in cells without the LPS challenge. In contrast, when PBMCs isolated 1 h after the intake of XN-rich hop extract were challenged with LPS, protein levels of IL-1β IL-6 and TNF-α in cell supernatant were not significantly different from those without the LPS challenge (Figure 3a–c). Furthermore, when being challenged for 6 h IL-1β, IL-6 and TNF-α protein levels in cell culture supernatant were still higher in LPS-stimulated cells isolated after the ingestion of the placebo than when subjects had consumed the XN enriched beverages; however, differences were less pronounced (Supplementary Figure S1). As protein levels of the three cytokines determined in untreated cells after the ingestion of the placebo and the XN-rich hop extract, respectively, were similar, only those measured after the intake of the placebo are shown.

### 2.3. Effect of the Oral Intake of XN on MD-2, TLR4 and sCD14 Protein in PBMCs

**Figure 4:**
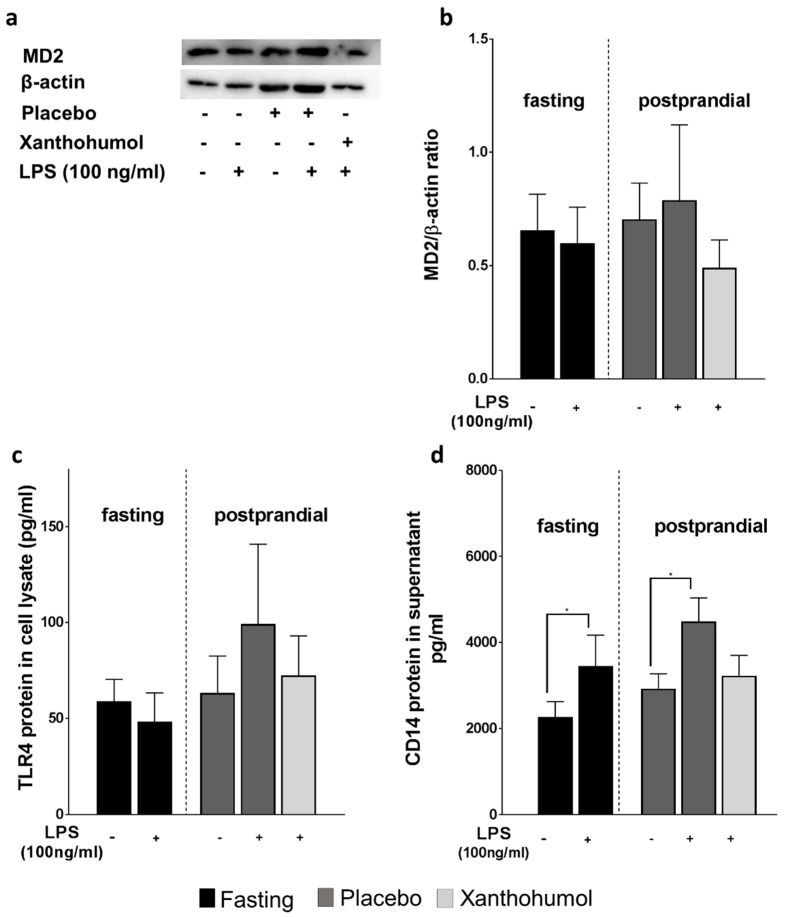
Protein concentration of MD−2, TLR4 and CD14 in LPS−stimulated PBMCs obtained from healthy study participants. Representative blots (**a**) and densitometric analysis of MD−2 western blot (**b**), TLR4 protein concentration in total protein lysate (**c**) and sCD14 protein concentration in cell culture supernatant (**d**) of PBMCs obtained from study participants either receiving a placebo or XN stimulated with LPS (100 ng/mL) for 6 h. LPS, lipopolysaccharide; MD−2, myeloid differentiation factor 2; PBMC, peripheral blood mononuclear cell; sCD14, soluble cluster of differentiation 14; TLR, toll−like receptor; XN, xanthohumol derived through a XN−rich hop extract. Data are expressed as means ± SEM. * = *p* < 0.05.

As it has been reported before that XN may dampen the LPS-dependent TLR4-response of immune cells through MD-2-dependent mechanisms [13,17], we next determined MD−2 and TLR4 protein levels in cells and sCD14 in cell supernatant. Neither protein levels of MD-2 nor of TLR4 were altered by the LPS-challenge of cells isolated before or after ingestion of the placebo and the XN enriched beverage, respectively (Figure 4a–c). In contrast, protein levels of sCD14 were significantly higher in LPS challenged cells isolated before the consumption of the beverages and those isolated 1 h after the intake of the placebo. In contrast, concentrations of sCD14 protein were unchanged in cell supernatant of LPS-challenged cells isolated after the intake of XN (Figure 4d).

### 2.4. Effect of XN on the LPS-Dependent Activation of TLR4 and the Effect of sCD14 Herein

**Figure 5:**
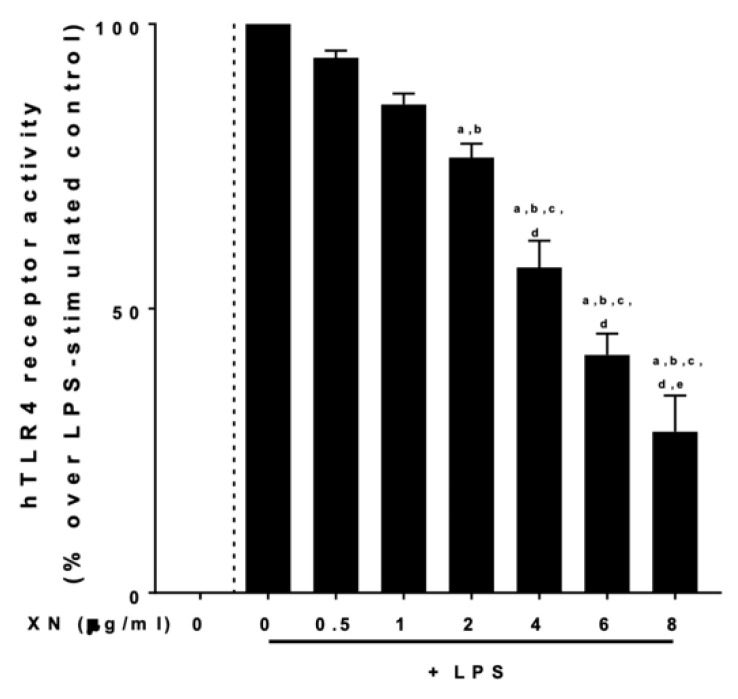
Receptor activities of HEK293 cells co-stimulated with LPS and XN for 12h. HEK293 cells were stimulated with LPS (100 ng/mL) and increasing concentrations of XN (0–8 μg/mL) for 12 h. LPS, lipopolysaccharide; XN, xanthohumol derived through a XN-rich hop extract. Data are expressed as means ± SEM. ^a^ = *p* < 0.05 compared to 0 XN + LPS, ^b^ = *p* < 0.05 compared to 0.5 XN + LPS, ^c^ = *p* < 0.05 compared to 1 XN + LPS, ^d^ = *p* < 0.05 compared to 2 XN, ^e^ = *p* < 0.05 compared to 4 XN + LPS.

**Figure 6:**
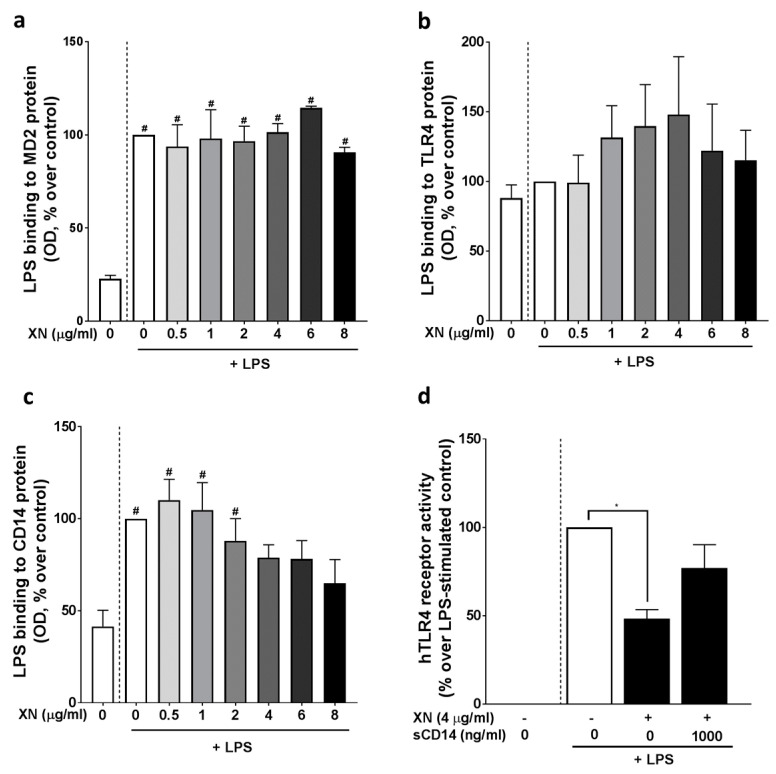
Inhibitory effect of XN on LPS−binding to MD−2, TLR4 and CD14. Effect of increasing concentrations of XN (0–8 μg/mL) on LPS−binding to MD−2 (**a**), TLR4 (**b**) and CD14 (**c**) as well as receptor activity of hTLR4 HEK293 cells co−stimulated with LPS (100 ng/mL), XN (4 μg/mL) and sCD14 (1000 ng/mL) for 12 h (**d**). LPS, lipopolysaccharide; MD−2, myeloid differentiation factor 2; sCD14, soluble cluster of differentiation 14; TLR, toll−like receptor; XN, xanthohumol derived through a XN−rich hop extract. Data are expressed as means ± SEM. # = *p* < 0.05 compared to unstimulated control (0 ng/mL LPS, 0 μg/mL XN). * = *p* < 0.05 compared to LPS−stimulated cells.

To further delineate mechanisms underlying the suppressive effects of XN on the LPS-dependent activation of PBMCs, we next determined if XN derived through a XN-rich hop extract alters the LPS-dependent activation of a commercially available HEK blue cells assay, in which cells are transfected with human TLR4, MD-2 and CD14. Results are shown in Figure 5. The XN-rich hop extract attenuated the activation of cells in an almost dose-dependent manner, with 4 μg/mL of XN diminishing the LPS-dependent activation of cells by ~50%. To further determine if the suppressive effects on TLR4 signaling were related to an interaction of the hop compound with MD-2, CD14 or TLR4, we adapted the in vitro assay of Chen et al. [21]. None of the doses of XN (0–8 μg/mL) used affected the binding of biotinylated LPS to MD-2 (Figure 6a) or TLR4 (Figure 6b). In contrast, the binding of biotinylated LPS to CD14 was dose-dependently inhibited by XN (Figure 6c). Furthermore, when stimulating the TLR4, MD-2 and CD14 transfected HEK blue cells with increasing sCD14 concentrations in the presences of 0 or 4 µg/mL XN and 0 or 100 ng/mL LPS, the ~50% suppression of the LPS-dependent activation of cells was attenuated when 1000 ng/mL sCD14 were added to the cell media (Figure 6d).

## 3. Discussion

Infections caused by bacteria are common and can lead to severe and even life-threatening health condition frequently demanding extended and cost-intensive treatments. Antibiotics are still the treatment option of choice for most bacterial disease. However, antibiotic resistances are increasingly often limiting therapeutic options [22]. Here, we determined the effect of an acute ingestion of low doses of a XN-derived through a XN-rich hop extract on the LPS-dependent immune responses in isolated PBMCs of healthy young women. The dose of XN used in our study was based on the average XN amount in brewing of ~250 mL beer [19,20] and on studies employing a co-cultural model to mimic the gut/blood barrier, where a clear permeation of XN through a Caco-2 cell monolayer and binding to immune cells was shown when cells were exposed to 0.125 mg of XN. Somewhat in line with the findings of others [10,23], we found that XN rapidly crosses the intestinal barrier here mimicked by Caco-2 cells and binds or is taken up by some cells in the PBMC fraction. Recently, we showed, that XN predominantly binds to the monocyte/ macrophage fraction of PBMCs, while no binding to T- and B-cells were shown in these experiments [14]. Furthermore, while using lower doses than other groups when showing anti-inflammatory effects in mice and rats [11,24,25], in the present study, LPS-dependent activation of PBMCs was dampened by XN derived through a XN-rich hop extract when compared to the placebo. These results are in line with recent findings of our own group showing that and oral intake of XN in doses alike can diminish the LTA-dependent immune responses of PBMCs in humans [14]. Still, the immune response was not completely diminished to that of unstimulated cells but rather, it was dampened by ~43–64% after 2 h and ~17–20% after 6 h, depending on the cytokine measured. Others have suggested before that a total suppression of immune responses to viral or bacterial challenges may be deleterious and may even worsen the severity of the disease and extend recovery [26]. Therefore, a dampening of the immune response may even be more wishful than a total abolishment of the immune response. Taken together, our results suggest that XN derived through a XN-rich hop extract when consumed at low doses may dampen the LPS-dependent immune response of PBMCs in healthy and normal weight women. However, as in the present study PBMCs were exposed to LPS only ex vivo after one acute ingestion of the hop compound, further studies are needed to determine if (1) effects alike are also present in humans suffering from an infection with Gram-negative bacteria and (2) when the compound is consumed repeatedly and (3) when 100% pure XN is used.

Through which mechanisms does XN dampen LPS-dependent activation of PBMCs?

The mechanisms involved in the uptake of XN, and if blood cells bind or take up the hop compound, are still not fully understood. Results of other groups have suggested before that XN may bind to MD-2 and may thereby interfere and dampen endotoxin-dependent activation of TLR4 in immune cells such as monocytes or macrophages [16]. Contrasting these findings, in the present study, while blocking LPS-dependent activation of HEK blue cells transfected with TLR4, MD-2 and CD14, neither LPS binding to MD-2 nor protein levels of MD-2 in LPS stimulated PBMCs were altered by the presence of XN. In contrast, we found that XN, almost in a dose-dependent manner, inhibited the bindings of biotinylated LPS to CD14 in a cell-free in vitro assay and diminished the release of sCD14 from LPS-stimulated PBMCs. Furthermore, sCD14 attenuated the inhibitory effects of XN derived through a XN-rich hop extract on the LPS-dependent activation of HEK blue cells transfected with TLR4. Interestingly, similarly to MD-2, XN derived through the XN-rich hop extract had no effect on TLR4 protein or LPS binding to TLR4. Somewhat in line with these findings, we recently reported that XN also attenuated the LTA-dependent stimulation of TLR2-signaling and that the protective effects of XN on the LTA-dependent activation of TLR2 seemed also to depend upon its effects on CD14 [14]. Furthermore, results of several studies have shown that LPS is bound to CD14 and then transferred to TLR4/MD-2 [27]. It further has been shown that CD14 is critical for the recognition of LPS by TLR4 and MD-2 [28,29]. In addition, other results suggest that CD14 is ”shedded” from the cell surface [30] and that this may be critical in the inflammatory response to bacterial toxins [31]. In summary, our data suggest that XN derived through a XN-rich hop extract dampens the LPS-dependent activation of the TLR4 signaling cascade through CD14-dependent mechanisms. Still, our results by no means preclude that XN may also affect other (intra) cellular signaling cascades, especially, when consumed at higher doses and/ or over an extended period of time. Rather, our results suggest, that when consumed at non-pharmacological concentrations, XN derived through a XN-rich hop extract may at least temporally attenuate the LPS/CD14-dependent activation of the TLR4-signaling cascade in blood immune cells.

### Limitations

When interpreting the data of the present study, some limitations need to be considered. For one, all cell stimulation experiments were carried out ex vivo with cells stemming only from healthy, normal weight women. Therefore, additional studies need to assess if these effects are also found in patients infected with Gram-negative bacteria. Furthermore, here, XN was only eaten once with PBMCs being isolated 1 h after the intake. Accordingly, from the presented data, no estimations regarding persistence and long-term effects of XN on bacterial toxin-triggered immune response can be made. Furthermore, in the present study, for ethical and safety reasons, XN was presented to study participants in form of a commercially available hop extract containing 75% XN but also other hop compounds (e.g., prenylflavonoids and geranyl flavonoids). It has been shown before by others that these by-products are so low in concentration, that physiological effects are considered unlikely [32]. To be consistent within the study, the same extract used in the human intervention was also employed in the cell culture experiments. In addition, in the present study, we only assessed effects in healthy, normal weight women. If effects alike are also found in overweight and/or older individuals as well as male subjects needs to be determined in future studies. Another limitation that needs to be taken in consideration is the use of a commercially available transfected HEK blue cell system to assess the effects of XN derived through the XN-rich hop extract on CD14/MD−2/TLR4 signaling. Indeed, while these cells are transfected with CD14, MD−2 and TLR4 it cannot be ruled out that this cell culture system does not resemble all molecular interactions found in isolated immune cells and in vivo, respectively. Moreover, in the present study the blockage of LPS-binding to CD14 by XN derived through the XN-rich hop extract was only shown in a cell-free in vitro assay bearing some limitations when extrapolating results to the in vivo situation.

## 4. Materials and Methods

### 4.1. Co-Culture Cell Assay

To determine the uptake and binding of XN to PBMCs, a co-culture model of Caco-2 cells and human PBMCs, isolated from buffy coat was employed (also see Figure 1a for study design). In brief, Caco-2 cells were grown according to the instructions of the manufacturer in a humidified, 5% carbon dioxide atmosphere to 100% confluence in semipermeable transwells by using DMEM medium containing 10% fetal bovine serum (FBS) (Pan-Biotech GmbH, Aidenbach, Germany), 100 μg/mL streptomycin and 100 U/mL penicillin (Pan-Biotech GmbH, Aidenbach, Germany). Integrity of Caco-2 monolayer was checked daily for 9 days by transepithelial electrical resistance (TEER). Once the Caco-2 cell monolayer had reached confluency and TEER was stable (day 9), PBMCs isolated from buffy coats of healthy donors were isolated by gradient centrifugation as detailed before by others [33] and were seeded in the basolateral compartment below the transwell containing the differentiated Caco-2 cells. Caco-2 cells were then incubated with XN (0–0.750 mg/mL) derived from a XN-rich hop extract for 1 and 2 h. Cell culture medium was collected in the basolateral compartment and PBMCs were washed, fixed on glass slides via cytospin and stained with DAPI (Sigma-Aldrich Corp., St. Louis, MO, USA). DAPI staining was detected using DAPI filter system included in the microscope (Leica Camera AG, Wetzlar, Germany). Binding of XN to cells was determined as detailed before by others [34,35]. In brief, spotted cells were excited at 480/40 nm and emission was detected at 530 nm to detect autofluorescence of XN.

### 4.2. Study Participants

Based on sample size calculations assuming 1–2 drop-outs, a total of 12 normal weight healthy female subjects were enrolled in the study. As the yield of PBMCs for the ex vivo stimulation experiments was insufficient at some time points, three of the subjects had to be excluded from the final analysis. Previous studies of others [36] assessing the reduction in pro-inflammatory cytokine release in human PBMCs were used to estimate the sample size via power analysis (a priori) (Gpower, Version 3.1.9.2). Only participants not reporting food intolerances or food allergies that would require a particular dietary intervention were enrolled. All study participants confirmed the absence of metabolic diseases, chronic inflammatory diseases or viral and bacterial infections within the last 3 weeks before the study. Furthermore, the use of anti-inflammatory medication was defined as exclusion criteria for the study. The study, which is part of a larger project assessing the acute and chronic effect of the intake of hop compounds such as XN and iso-α-acids on the immune response of bacterial-toxin-activated PBMCs (ClinicalTrials.gov: NCT04847193), was approved by the Ethics Committee of the University of Vienna, Vienna, Austria (00367) and was carried out in accordance with the ethical standards laid down in the Declaration of Helsinki of 1975 as revised in 1983.

### 4.3. Intervention Study

Before the intervention the participants underwent a wash-out phase for 14 days during which they refrained from all hops containing products such as beer. After the wash-out, participants were then randomly and single-blinded assigned to receive a study drink containing 10 mL water, thickener (Nestlé S.A., Vevey, Switzerland), 70 mg skim milk powder, and lemon flavor (Pepsico Inc., Purchase, NY, USA) enriched with 0.125 mg XN (XanthoFlav, generous gift from Hopsteiner GmbH, Au an der Hallertau, Germany) or a placebo. XanthoFlavTM consists of XN (75%) and other prenylated flavonoids (<25%) occurring naturally in hops (for further details, also see: https://www.hopsteiner.com/wp-content/uploads/2021/12/26_21_ls_xanthoflav.pdf, accessed on 10 August 2022). Among these prenylated flavonoids kaempferol-3-O-β-D-glucopyranosid, cis-/trans-p-coumaric acid methylester and n-multifidol-di-C-glucopyranoside are the quantitively most common flavonoids next to XN. However, concentrations are very low and only reliably verifiable by LC-MS [33]. The placebo was similar to the study drink but lacked the addition of XN. The study drink was consumed within 15 min in combination with a standardized breakfast containing 2 medium sized pretzels and 30 g butter. Before, in fasted state, and 60 min after the intake of the study drink, blood was collected, and PBMCs were isolated as detailed below. After a second wash-out phase lasting at least 7 days, in which the participants were again asked to refrain from all hops containing foods and beverages, the intervention was repeated in a cross-over design.

### 4.4. Isolation and Culture of PBMCs

Following the manufacturer instructions, a Vacutainer^®^ CPTTM System (Becton Dickinson GmbH, Heidelberg, Germany) was used to isolate PBMCs from whole blood samples acquired from each participant at fasted state and after the consumption of the combination of study drink and the standardized breakfast. Isolated PBMCs were then cultivated in RPMI-1640 medium (Sigma-Aldrich Corp., St. Louis, MO, USA) with 10% fetal bovine serum (Pan-Biotech GmbH, Aidenbach Germany), 100 μg/mL streptomycin and 100 U/mL penicillin (Pan-Biotech GmbH, Aidenbach, Germany) for 1 h. Subsequently, cells were either challenged with 0 or 100 ng/mL LPS for 2 h and 6 h, respectively. Cell culture supernatant was collected and PBMCs were lysed using RIPA buffer to obtain total protein. Supernatant and protein were stored at −80 °C until further use.

### 4.5. Cell Culture Experiments with hTLR4 Transfected HEK Cells Response

To assess the effects of XN on CD14/TLR4 signaling, a commercially available reporter gene assay with HEK-BlueTM TLR4 cells, co-transfected with human TLR4, human MD-2 and human CD14 as well as an inducible secreted embryonic alkaline phosphatase (SEAP) fused to nuclear factor ‘kappa-light-chain-enhancer’ of B-cells (NF-κB) and activator protein 1 (AP-1) was used (InvivoGen, CA, USA, Cat.Number: hTLR4 = hkb-htlr4). Following the instructions of the manufacturer, cells were grown in a humidified, 5% carbon dioxide atmosphere and were grown up to 80% confluence using DMEM media (Pan-Biotech, GmbH, Aidenbach, Germany). In a first set of experiments, cells were challenged with 100 ng/mL LPS for 12 h in the presence of 0–8 µg/mL XN derived through the XN-rich hop extract. Activity of TLR4 was indirectly determined by measuring SEAP induced color change of cell culture medium at 655 nm. In a second set of experiments, cells were again grown to 80% confluence and challenged with 0 or 100 ng/mL LPS in the presence of 0 or 4 µg/mL XN and 0–1000 ng/mL sCD14. The XN dose used in this experiment was determined in the first set of experiments. After 12 h, color changes of medium were determined at 655 nm.

### 4.6. Western Blot

RIPA buffer was used to extract total protein from cells as detailed before [37]. Protein extracts and cell culture supernatant, respectively, were separated in a 10% polyacrylamide gel and were transferred on a polyvinylidene difluoride membrane (Bio-Rad Laboratories, Hercules, CA, USA). Membranes were blocked with either 3% skim milk powder or 5% BSA for 1 h prior to the incubation with a specific monoclonal antibody for MD-2 (#NB100-56655; Novus Bio, Centennial, CO, USA) followed by a 1 h incubation with an appropriate secondary antibody (#7074S for MD-2, Cell Signaling Technology Inc., MA, USA). Bands were detected using Super Signal West Dura kit (Thermo Fisher Scientific, Waltham, MA, USA). Densitometric analysis of bands was performed using Image Lab 6.0 Software (Bio-Rad Laboratories, Hercules, CA, USA).

### 4.7. Enzyme-Linked Immunosorbent Assays (ELISA’s)

IL-1β, IL-6, TNF-α, TLR4 and sCD14 protein concentrations were analyzed in cell culture supernatant and extract from total protein using commercially available ELISA kits (IL-1β: Sigma-Aldrich Corp., St. Louis, MO, USA; IL-6: Bio-Techne Corp., Minneapolis, MN, USA; TNF-α: BioVendor R&D^®^, Czech Republic; CD14: Sigma-Aldrich Corp., St. Louis, MO, USA).

### 4.8. LPS Binding Assays

To determine if XN binds to MD-2, CD14 or TLR4, a LPS binding assay adapted from Zhang et al. was used [38]. In brief, polystyrene 96 well plates (R&D Systems Inc., Minneapolis, MN, USA) were coated with MD-2 (#NB100-56655; Novus Bio, Centennial, CO, USA), CD14 (#56082S, Cell Signaling Technology Inc, Danvers, MA, USA) or TLR4 (#NB100-56723SS; Novus Bio, Centennial, CO, USA) antibodies over night at 4 °C. Plates were blocked with 3% BSA for 2 h followed by an incubation with their respective recombinant protein (MD-2: 1787-MD, Bio-Techne Corp., Minneapolis, MN, USA; CD14: 110-01, PeproTech, Cranbury, NJ, USA; TLR4: 160-06, PeproTech, Cranbury, NJ, USA) (1 μg/mL) for 1.5 h. XN in concentrations ranging from 0–8 μg/mL derived through the XN-rich hop extract was added to the plate in presence or absence of LPS-biotin (50 ng/mL, InvivoGen, CA, USA,) for 1 h. After a 1 h incubation with HRP-Streptavidin (Bio-Techne Corp., Minneapolis, MN, USA) plates were incubated with TMB (Thermo Fisher Scientific, Waltham, MA, USA) for another 15 min. Excess antibody and streptavidin-HRP solution was removed by washing with 0.05% PBST. To stop the reaction, 2 N H_2_SO_4_ was added to the plate and absorbance was measured at 450 nm.

### 4.9. Statistical Analyses

Data are presented as means ± standard error of the means (SEMs). The Friedman test with Dunn’s multiple comparison test and the Wilcoxon test was used to determine statistically significant differences between interventions and one-way ANOVA was used for all other comparisons (GraphPad Prism Software, CA, USA). *p* ≤ 0.05 was selected as the level of significance.

## 5. Conclusions

Taken together, the results of our study suggest that the acute consumption of low doses of XN derived through a XN-rich hop extract may dampen the LPS-dependent immune response of PBMCs in healthy women. Our results further suggest that the beneficial effects of the hop compound are related to an inhibition of the binding of LPS to CD14. While in the present study it was shown that the immunosuppressed effects of XN are found within 1 h after the oral intake of XN derived through a XN-rich hop extract, further study is needed to determine dose- and time-responses as well as the exact mechanisms underlying the inhibitory effects of XN.

## Figures and Tables

**Table 1:** Anthropometric and health characteristics of study participants. Data are expressed as means ± SEM. AST, aspartate amino transferase; ALT, alanine amino transferase; CRP, c reactive protein; γ-GT, γ-glutamyltransferase; HDL, high density lipoprotein; and LDL, low density lipoprotein.

Parameter	Healthy Participants
Sex (m/f)	0/12
Age (years)	26.1 ± 1.1
Body weight (kg)	60.9 ± 2.1
Height (m)	1.66 ± 0.02
BMI (kg/m^2^)	22.1 ± 0.5
Blood pressure	
Systolic (mmHg)	126.6 ± 7.57
Diastolic (mmHg)	78.3 ± 3.6
Fasting glucose (mg/dL)	90.1 ± 2.2
Uric acid (mg/dL)	4.1 ± 0.2
AST (U/L)	29.8 ± 8.7
ALT (U/L)	33.7 ± 16.6
γ-GT (U/L)	12.2 ± 1.2
Cholesterol (mg/dL)	171.8 ± 8.4
HDL cholesterol (mg/dL)	66.4 ± 4.8
LDL cholesterol (mg/dL)	91.6 ± 6.1
Triglycerides (mg/dL)	77.6 ± 9.2
CRP (mg/dL)	0.03 ± 0.03

## Supplementary Materials

The following supporting information can be downloaded at: https://www.mdpi.com/article/10.3390/ijms232012702/s1.
