# Supplementary material for: A Xanthohumol-Rich Hop Extract Diminishes Endotoxin-Induced Activation of TLR4 Signaling in Human Peripheral Blood Mononuclear Cells: A Study in Healthy Women
Source: Int J Mol Sci. 2022 Oct 21;23(20):12702. doi: 10.3390/ijms232012702 (PMC9603845; doi:10.3390/ijms232012702)
Supplement: Supplementary file 1 [file ijms-23-12702-s001.zip › ijms-1918506-supplementary.pdf]

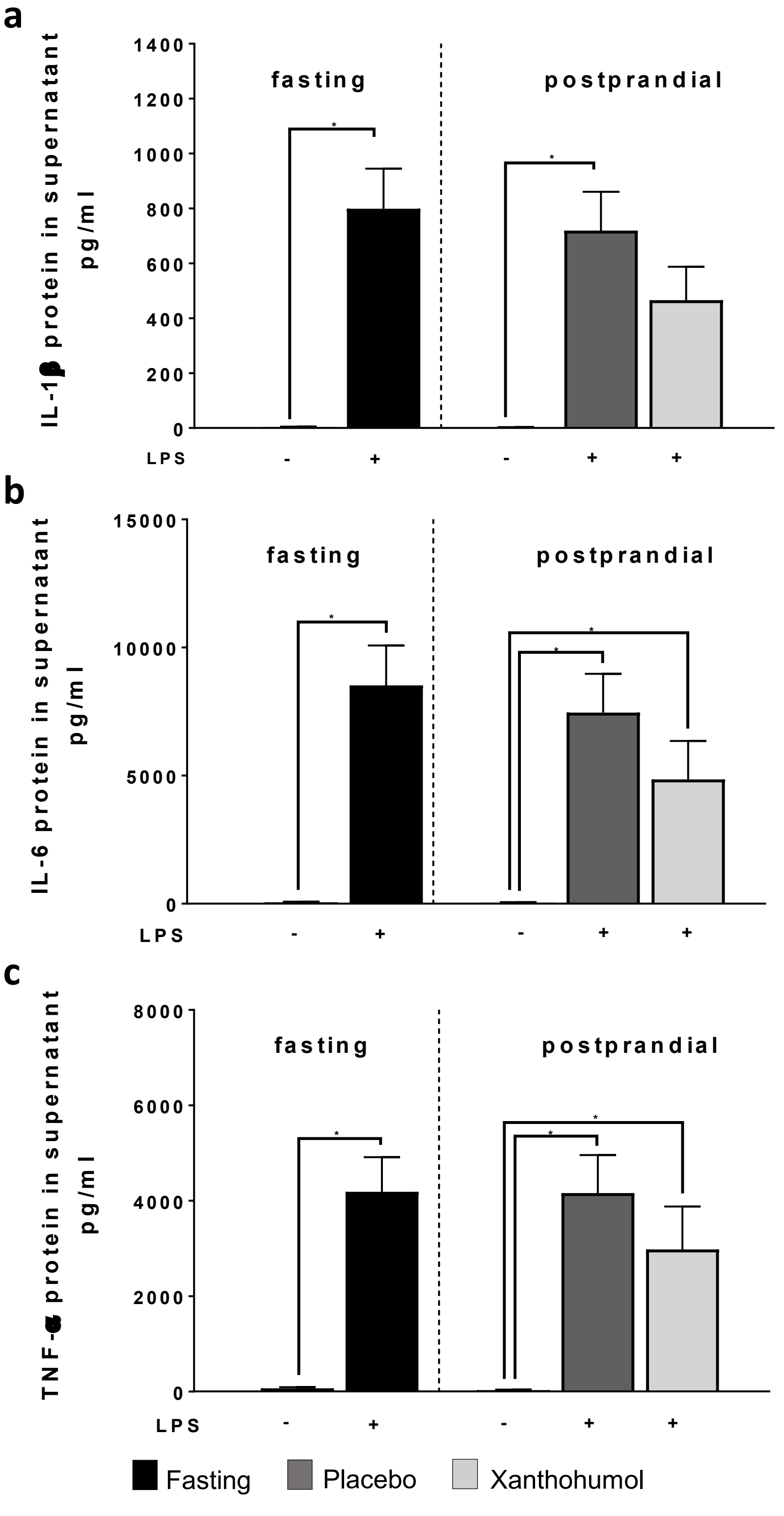


**Figure S1.** Cytokine concentrations in supernatant of LPS-stimulated PBMCs obtained from healthy study participants. Protein concentrations of IL-1β (a), IL-6 (b) and TNF-α (c) in cell culture supernatant of PBMCs stimulated with 0 or 100 ng/ml LPS for 6 h isolated from healthy study participants receiving either a placebo or the study drink containing XN. IL, interleukin; LPS, lipopolysaccharide; PBMC, peripheral blood mononuclear cell; XN, xanthohumol. Data are expressed as means ± SEM. * = $p < 0.005$.
